# Development of a Carvedilol-Loaded Solid Self-Nanoemulsifying System with Increased Solubility and Bioavailability Using Mesoporous Silica Nanoparticles

**DOI:** 10.3390/ijms26041592

**Published:** 2025-02-13

**Authors:** Hangeul Jang, Nahyun Kim, Sung Giu Jin

**Affiliations:** Department of Pharmaceutical Engineering, Dankook University, 119 Dandae-ro, Dongnam-gu, Cheonan 31116, Republic of Korea; gksruf132@gmail.com (H.J.);

**Keywords:** mesoporous silica nanoparticles, carvedilol, solid self-nanoemulsifying drug delivery system

## Abstract

This study developed a solid self-nanoemulsifying drug delivery system (S-SNEDDS) to improve the oral bioavailability of poorly soluble carvedilol using mesoporous silica nanoparticles (MSNs). The liquid self-nanoemulsifying drug delivery system (L-SNEDDS) consisted of carvedilol, Peceol, Tween 80, and Labrasol in a weight ratio of 10:25:50:25. The liquid SNEDDS was suspended in MSN at various ratios and spray-dried to produce S-SNEDDS. The emulsion size, PDI, solubility, and dissolution of various ratios of MSN were evaluated to make the optimal S-SNEDDS. The optimal S-SNEDDS, manufactured using a ratio of MSN to L-SNEDDS 1000 at 500, formed a nanoemulsion and achieved efficient supersaturation compared to carvedilol alone, which significantly improved drug solubility (approximately 400 times), dissolution (approximately 5.7 times at 60 min), area under the curve (AUC) (21.7 times), and maximum plasma concentration (Cmax) (15.7 times). In addition, the physicochemical properties of the optimal S-SNEDDS were evaluated by differential scanning calorimetry (DSC), X-ray powder diffraction (XRD), Fourier transform infrared (FT-IR), particle size, and scanning electron microscopy (SEM) images. S-SNEDDS showed a smaller particle size than MSN alone, and the crystalline drug was transformed into an amorphous substance, resulting in encapsulation in MSN. These results suggest that MSN can be a novel biocompatible carrier contributing to a safer and more effective delivery system.

## 1. Introduction

Carvedilol is a non-selective β-blocker with additional adrenergic receptor α1 blocking activity and is widely prescribed to manage hypertension, heart failure, and angina pectoris [1]. Although carvedilol has therapeutic efficacy, it is a class II drug in the biopharmaceutical classification. It has poor water solubility and low bioavailability, making it challenging to formulate and limiting its absorption and therapeutic effect. These characteristics may hinder clinical application, as patients may need higher doses or more frequent administration to achieve the desired effect, which may increase the risk of adverse effects [2].

Therefore, improving the solubility and bioavailability of drugs belonging to the BCS class II, such as carvedilol, is essential for optimizing therapeutic benefits. Various solubilization techniques have been developed to enhance the bioavailability of active drugs with limited solubility. These include particle size reduction techniques such as micronization and nanonization to increase the surface area, enhancing solubility [3,4]. Amorphous solid dispersions enhance solubility by inhibiting crystallization and creating supersaturated solutions. Polymeric carriers in solid dispersions and cyclodextrin complexes are also widely adopted to improve the solubility and stability of hydrophobic drugs [5]. Another strategy uses carriers and unique delivery systems to improve solubilization [6]. Lipid-based formulations such as SNEDDS are one of the most effective methods to enhance the bioavailability of hydrophobic drugs [7]. Encapsulation in liposomes, nanoparticles, and micelles can improve the solubility of poorly soluble drugs in aqueous environments [8]. SNEDDS are isotropic mixtures of oils, surfactants, and co-surfactants that spontaneously form nano-sized emulsions when exposed to gastrointestinal fluids. These systems enhance drug dissolution and absorption by providing a large surface area for drug release, increasing solubility, improving mucosal permeability, and improving overall bioavailability [9].

S-SNEDDS offers additional benefits by incorporating solid-state properties to improve the formulation’s stability, handling, and portability. Compared with conventional L-SNEDDS, S-SNEDDS can improve the drug’s physical and chemical stability, minimize leakage problems during storage and transportation, and control drug release, potentially improving dosing convenience. Solidification techniques such as spray drying, freeze drying, and adsorption onto solid carriers can convert L-SNEDDS into more convenient dosage forms, such as powders or tablets, while preserving their self-emulsifying properties to aid absorption [10].

The choice of solid carrier is critical for developing effective S-SNEDDS, as it directly affects the drug stability, release profile, and bioavailability. An ideal carrier should have the properties to ensure high drug loading, maintain the nano-emulsification potential of SNEDDS during reconstitution, and stabilize the drug and excipients within the solid matrix [11]. In particular, porous carriers are highly desirable, as they provide a large surface area for drug adsorption, which helps the uniform distribution of SNEDDS components and enhances the dissolution rate of drugs with low solubility [11].

MSN has been investigated as promising drug delivery systems due to their excellent biocompatibility and ideal porous structure for drug loading. They have also been used for drug delivery, such as anticancer drugs. In particular, the physicochemical properties of silica particles can be modified at the atomic level by introducing modifier cations into the Si–O network. For example, amorphous silica particles containing Ca^2+^, Na^+^, and phosphate have been used as excellent biodegradable biomaterials. Iron-doped silica particle nano-shells, Ca-doped silica nanoparticles, and manganese-doped silica nanoparticles have been used as magnetic resonance imaging contrast agents for anticancer drug delivery [12]. Recently developed zinc-doped silica-based mesoporous particles exhibit various bioactive activities [13]. In addition, S-SNEDDS using MSN can be used as a solubilizing carrier for insoluble substances. The high surface area and well-defined pore structure enable efficient loading of drug-SNEDDS mixtures. At the same time, the mesoporous nature can control the drug release by modulating the diffusion of encapsulated drug molecules. Therefore, using MSN particles in S-SNEDDS formulations can be a novel biocompatible carrier that enhances the solubility and bioavailability of carvedilol and contributes to an overall safer and more effective delivery system [14].

Compared with S-SNEDDS using conventional solid carriers, MSN can efficiently load drugs and be absorbed by the body. An important issue is determining the amount of MSN required to solidify L-SNEDSS and improve drug delivery efficiency [15,16].

**Figure 1:**
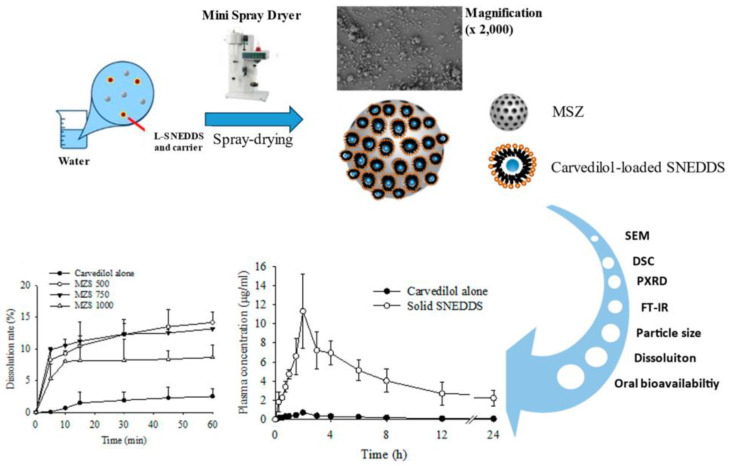
Schematic concept of carvedilol-loaded SNEDDS using MSN.

This study aimed to develop an S-SNEDDS formulation using carvedilol as a poorly soluble model drug to improve solubility and bioavailability using various ratios of MSN particles. L-SNEDDS was prepared using selected oils, surfactants, and co-surfactants, and S-SNEDDS was prepared using various MSN through a spray dryer. The optimal MSN ratio that provides the smallest nanoemulsion particle size and improves solubility and dissolution rate was selected. The pharmacokinetic profiles of the S-SNEDDS were compared in rats administered compared to the carvedilol alone. Moreover, the physicochemical properties were evaluated using DSC, XRD, FT-IR, and SEM. The conceptual framework of the study is illustrated in Figure 1.

## 2. Results and Discussion

### 2.1. Impact According to MSN Ratio

MSN can exhibit various bioactive activities and be utilized as drug-delivery carriers [17]. Zinc contained in MSN is essential for growth, healthy development, and numerous physiological functions [17]. The ion exhibits preferential antimicrobial effects and shows potential as an anticancer agent [18]. With these features, the study of S-SNEDDS using MSN can be utilized as a solubilizing carrier for poorly soluble substances and to deliver zinc ions’ properties. The high surface area and well-defined pore structure allow efficient loading of drug-SNEDDS mixtures and improve the flowability by loading the L-SNEDDS with hydrophobic properties, enabling solidification [19]. The MSN can provide a protective environment that prevents the crystallization of drugs, which can further improve the stability and bioavailability of poorly soluble drugs such as carvedilol. MSN contains SiO_2_ and ZnO (atomic composition: Si 30.4%, O 68.0%, Zn 1.6%). The average particle size of the used MSN is 250 nm, and the pore diameter and volume are 2.54 nm and 0.81 cm^3^/g, respectively. In addition, the surface area is 838 m^2^/g [20].

SNEDDS form fine nanoemulsions with gentle shaking to increase solubility and bioavailability when contacted with water [21]. Based on prior research findings, this study chose Peceol, Tween 80, and Labrasol as the oil, surfactant, and cosurfactant, respectively. L-SNEDDS was formulated with an optimized weight ratio of 25:50:25 [1]. S-SNEDDS was prepared by spray drying using MSN as a carrier and evaluated at various ratios to determine its solubility and dissolution properties, as shown in Table 1.

**Figure 2:**
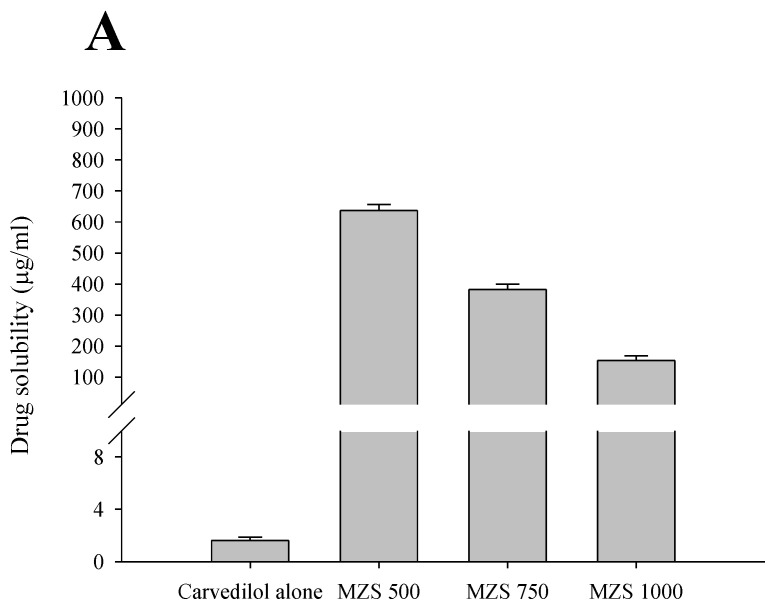

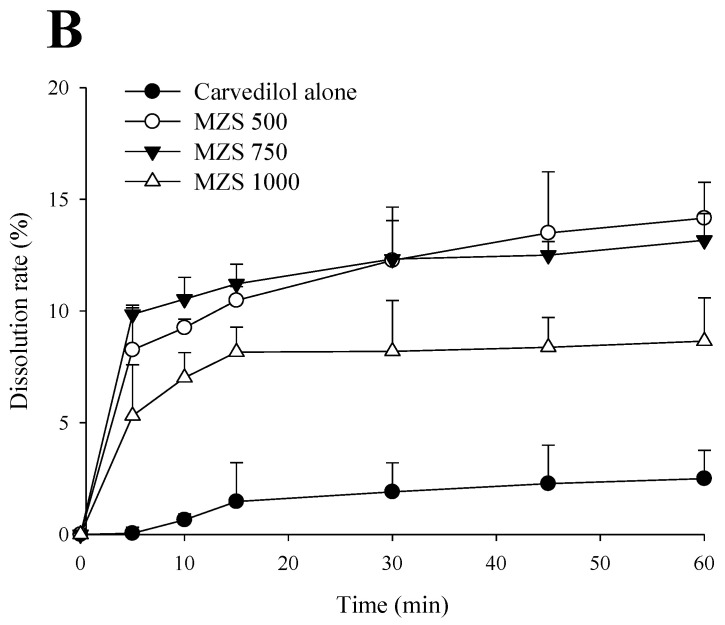
Aqueous solubility (**A**) and dissolution (**B**) of carvedilol. Each value represents the mean ± S.D. (n = 3).

S-SNEDDS was manufactured using MSN. MSN less than 500 mg was excluded because coagulated particles could not be obtained, and MSN more than 1000 mg was excluded because the total dose may be excessive due to the low drug ratio. The solubility and dissolution of S-SNEDDS according to the ratio of MSN were compared with that of the drug alone (Figure 2). S-SNEDDS manufactured using MSN all showed significantly increased solubility compared to the drug alone. However, the drug solubility decreased as the ratio of MSN increased (637.3 ± 19.0 vs. 382.1 ± 17.9 vs. 153.1 ± 16.1 mg/mL). MSN 500, which showed the highest solubility, showed a 400-fold solubility increase compared to the drug alone (637.3 ± 19.0 vs. 1.6 ± 0.3 mg/mL). The decrease in solubility as the MSN ratio increases suggests that MSN is an inorganic carrier exhibiting hydrophobic properties [22].

In addition, the dissolution test was performed according to the MSN ratio (Figure 2B). All S-SNEDDS showed an increased dissolution pattern compared to carvedilol alone. In addition, similar to the solubility results, MSN 1000, which had the highest MSN ratio, showed the lowest dissolution rate at 60 min. However, there was no significant difference between MSN 500 and MSN 750. Similar to the solubility results, the dissolution test results show a phenomenon in which the dissolution decreases as the ratio of MSN increases due to the hydrophobic characteristics of MSN [23].

**Figure 3:**
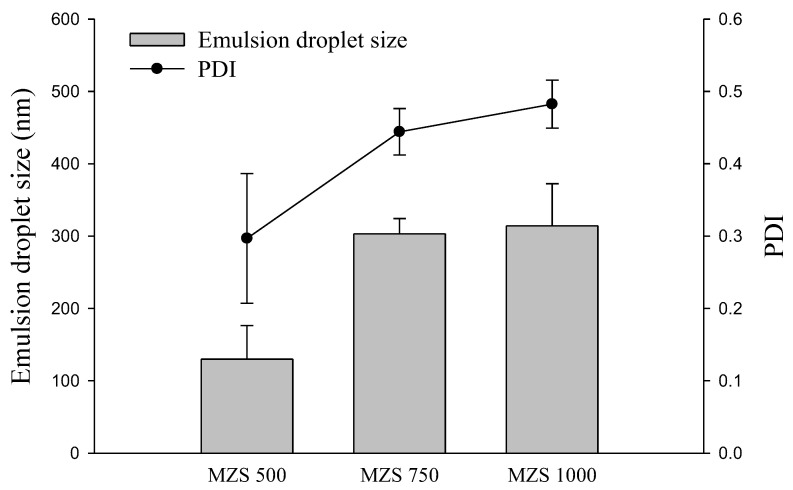
Impact of MSN ratio on droplet emulsion size and PDI of S-SNEDDS. Each value represents the mean ± S.D. (n = 3).

The emulsion droplet size is a critical parameter in SNEDDS, as it directly influences the dissolution capacity of poorly water-soluble drugs in aqueous environments. The smaller the emulsion droplets, the larger the surface area, which can lead to higher solubility and dissolution rate [24]. Figure 3 shows the average particle size and PDI values of the emulsion droplets obtained by redispersing S-SNEDDS. MSN 500 showed the smallest emulsion size (130.0 ± 46.3 nm) and PDI (0.297 ± 0.090). The zeta potential of MSN 500 was −32 mV, indicating a stable emulsion. This result is the basis for the highest solubility and dissolution results of MSN 500. In addition, this result indicates a small emulsion size compared to that of L-SNEDDS (approximately 250 nm) [1]. A stable and small emulsion size is essential in increasing the solubility and dissolution of poorly soluble drugs [25]. S-SNEDDS using MSN showed a 400-fold increase in solubility and a 5.7-fold increase in release (based on 60 min) compared to poorly soluble carvedilol alone.

**Figure 4:**
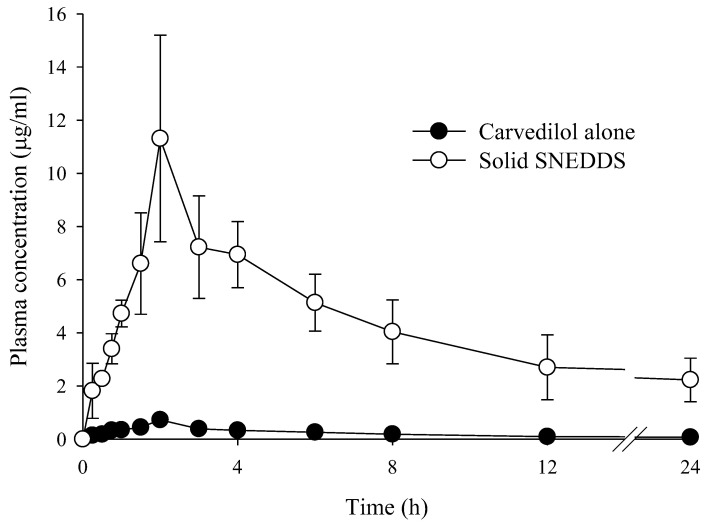
Plasma concentration-time profiles of carvedilol after oral administration of carvedilol alone and S-SNEDDS in rats. Each value represents the mean ± S.D. (n = 6).

Table 2 and Figure 4 show the plasma concentration changes and the corresponding pharmacokinetic parameters of carvedilol after oral administration of carvedilol alone and S-SNEDDS 40 mg/kg. MSN 500 was used because it showed the best solubility, dissolution rate, and emulsion particle size. Compared with carvedilol alone, S-SNEDDS showed significantly increased plasma concentrations at all time points. The enhanced solubility, dissolution, and reduced emulsion droplet size likely contribute to the higher plasma concentrations observed with S-SNEDDS. Compared with carvedilol alone, S-SNEDDS provided higher AUC (21.7-fold) and C_max_ (15.7-fold) values, indicating a significantly increased oral bioavailability. There was no significant difference in elimination constant (K_el_) and half-life (t_1/2_) between carvedilol alone and S-SNEDDS [2]. The enhanced oral bioavailability of carvedilol with S-SNEDDS can be attributed to the generation of supersaturated nano-sized emulsions in the gastrointestinal tract [26]. This result may imply an improved therapeutic effect of the drug. The use of MSN may pose a new challenge in drug delivery along with the solubilization effect of poorly soluble drugs.

### 2.2. Evaluation of Physicochemical Properties

**Figure 5:**
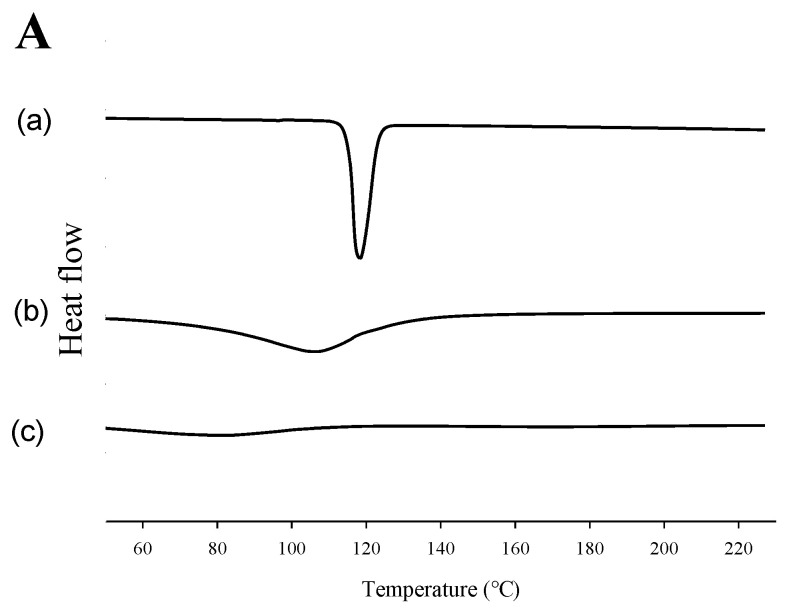

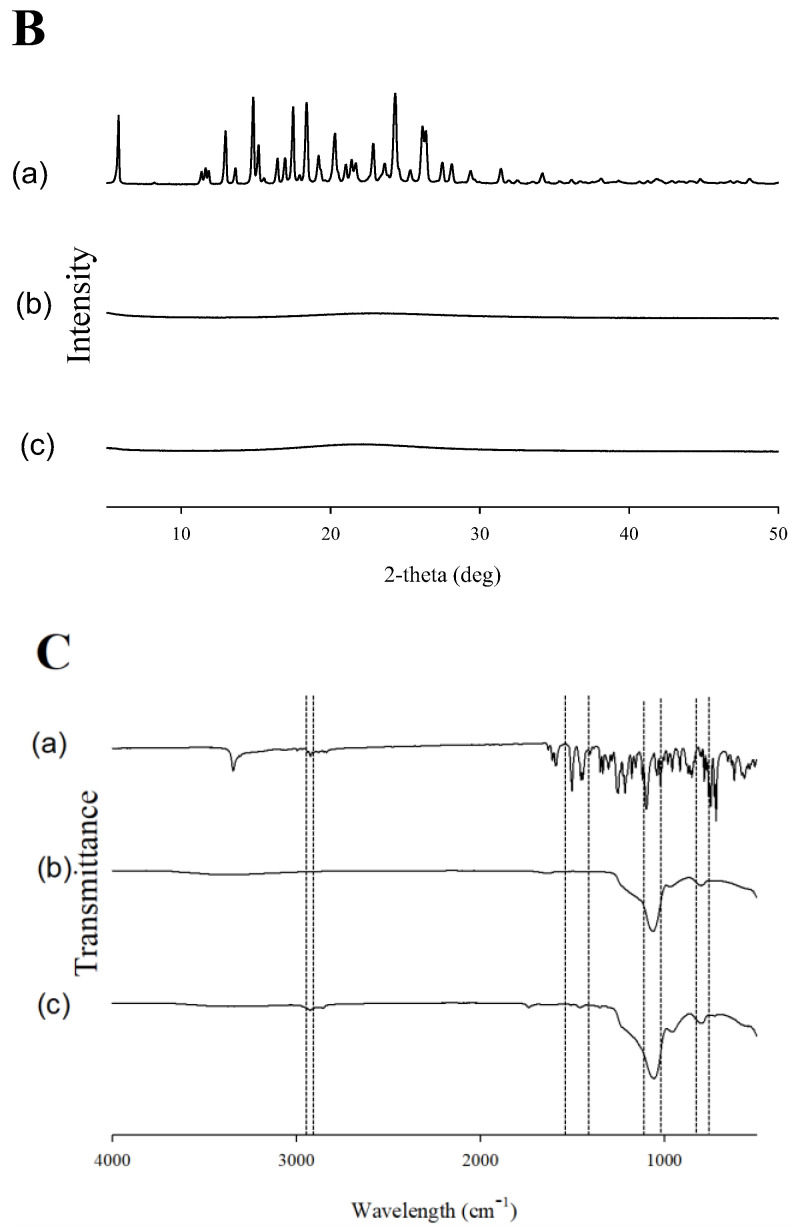
DSC thermograms (**A**), PXRD patterns (**B**), and FT-IR spectra (**C**): (a) carvedilol; (b) MSN; (c) S-SNEDDS.

The physicochemical characterization of S-SNEDDS (MSN 500) was evaluated using DSC, PXRD, and FT-IR (Figure 5). DSC analysis detected endothermic events, including melting, chemical decomposition, or phase transitions. The thermal aspects of carvedilol alone, MSN alone, and S-SNEDDS (MSN 500) are shown in Figure 5A. The DSC graph shows that carvedilol alone exhibited an endothermic peak near 120 °C. This result can be explained by the crystalline state and melting point of carvedilol alone (Figure 5A(a)). In addition, MSN alone (Figure 5A(b)) showed the result without any specific endothermic peak [27]. This result suggests that MSN exhibits an amorphous structure. When containing drugs, S-SNEDDS (MSN 500) (Figure 5A(c)) showed similar results to MSN alone. This result suggests that the carvedilol peak disappears in S-SNEDDS, indicating that crystalline carvedilol has been converted to an amorphous form. In addition, similar results to MSN alone may suggest that drug-loaded SNEDDS has been encapsulated in MSN [28].

Figure 5B shows the XRD pattern. XRD was evaluated to confirm the crystallinity of the drug and S-SNEDDS. Carvedilol alone (Figure 5B(a)) showed a unique crystalline pattern. MSN alone (Figure 5B(b)) showed the diffraction of MSN without sharp peaks [29]. This result implies that MSN has amorphous characteristics and an amorphous structure. However, S-SNEDDS (MSN 500) (Figure 5B(c)) showed that the unique pattern of carvedilol had disappeared. XRD analysis confirmed the transformation of carvedilol into an amorphous form within S-SNEDDS [30]. Consistent results from both DSC and XRD evaluations indicated that the crystalline structure of carvedilol was successfully converted to an amorphous state in S-SNEDDS. This result may imply that carvedilol loaded-SNEDDS was encapsulated within the MSN [31,32].

Changes due to chemical interactions of functional groups of components can be extracted through FT-IR spectra (Figure 5C). In the FT-IR spectrum of carvedilol alone, characteristic absorption bands at 2853 cm^−1^ (C-H aliphatic stretching), 1687 cm^−1^ (C=O aromatic stretching), and 1098 cm^−1^ (C-O stretching) were observed, which are consistent with the previously reported results (Pešić et al., 2021) [33]. Figure 5C(b) presents the FT-IR spectrum of MSN, with characteristic peaks at 1060 cm^−1^ and 790 cm^−1^ corresponding to the bending vibrations of the Si-O functional group [34]. The FT-IR spectrum of S-SNEDDS (Figure 5A(c)) using MSN when containing the drug is shown in Figure 5C(c). A comparison of Figure 5C(a,b) with Figure 5C(c) reveals no new peaks in the S-SNEDDS spectrum, suggesting that the interaction between carvedilol and MSN is due to physical bonding rather than a chemical reaction [35].

**Figure 6:**
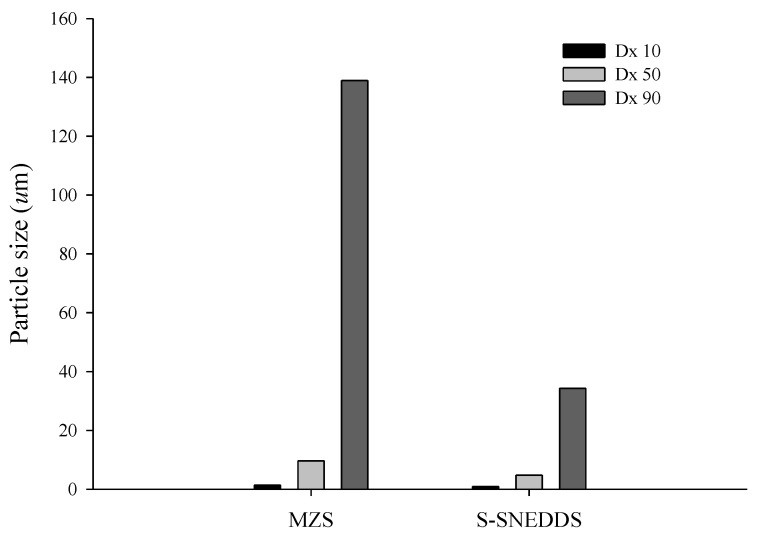
Particle size distribution of MSN and S-SNEDDS.

The particle sizes of MSN alone and S-SNEDDS (MSN 500) were compared using laser diffraction analysis (Figure 6). MSN alone produced a median size of 9.66 µm. In addition, S-SNEDDS showed a particle size of 4.82 µm. Since S-SNEDDS was spray-dried, it showed a significantly reduced particle size compared to MSN alone [36,37]. In addition, these particles were considerably more significant than the MSN alone particles (250 nm), indicating that L-SNEDDS exists in the form of aggregates as it solidifies.

**Figure 7:**
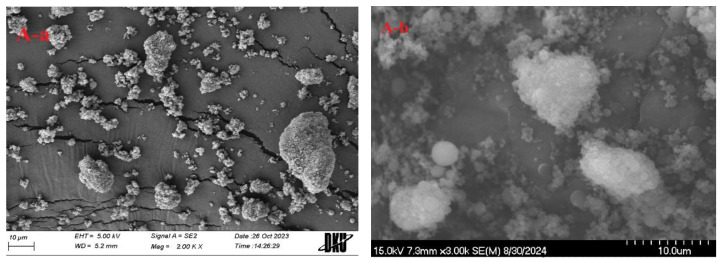

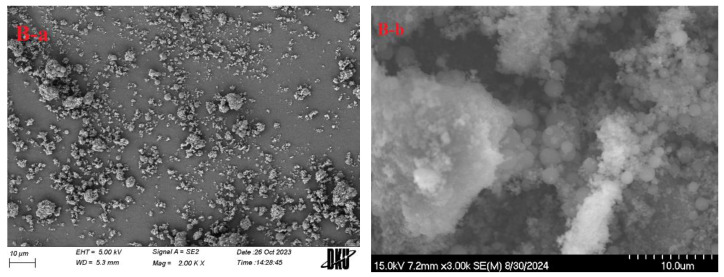
SEM photographs: (**A**) MSN, (**B**) S-SNEDDS; (a) (×2000), (b) (×3000).

The morphological structures of MSN alone and S-SNEDDS (MSN 500) are shown in the SEM images in Figure 7. Figure 7A shows the appearance of MSN with a porous surface with an irregular and wide size distribution [38]. In contrast, S-SNEDDS (Figure 7B) shows particles with a relatively narrow distribution and smaller particles than MSN particles [39]. These results demonstrate that carvedilol was encapsulated in L-SNEDDS and encapsulated in MSN. In addition, it also shows that it exists as aggregates. Overall, the trend of the SEM results is consistent with the particle size distribution. There was a significant size reduction in S-SNEDDS. Additionally, in line with the DSC and XRD findings, the SEM images did not reveal any crystalline drug. Instead, they displayed only the MSN carrier with small particle sizes, indicating that the drug was encapsulated within MSN and converted into an amorphous form.

## 3. Materials and Methods

### 3.1. Materials

MSN (SMB-7) was provided by CEN Co. (Miryang-si, Republic of Korea). Carvedilol was supplied by Cipla Ltd. (Mumbai, India). Peceol and Labrasol were supplied by Gattefossé (Saint-Priest, France). Tween 80 was purchased from Daejung Chemicals & Metals Co. (Siheung, Republic of Korea). All other chemicals and solvents used in this study were of reagent grade and were either used as received or further purified when necessary.

### 3.2. Animals

Twelve healthy male Sprague Dawley rats, 9 weeks old and weighing 280–320 g, were purchased from Koatech (Pyeongtaek, Republic of Korea). The animals were housed in a controlled environment maintained at 22 °C and a relative humidity of 45–60%. Before drug administration, the rats underwent a food-withholding period of 12–18 h, during which they had continuous access to water. All pharmacokinetic procedures and animal care complied with NIH guidelines, and the study protocol was approved by the Institutional Animal Care and Use Committee (IACUC) of Dankook University (Approval Number: DKU-20-014).

### 3.3. Preparation of S-SNEDDS

L-SNEDDS was prepared by dissolving carvedilol, Peceol, Tween 80, and Labrasol in an optimized mixture with a weight ratio of 10:25:50:25, using a vortex mixer until fully dissolved. The formulation was coagulated using a Büchi mini spray dryer (B-290; Meierseggstrasse, Switzerland). The spray drying conditions were established based on preliminary tests and previous optimization studies [1].

Before spray drying, 5 mL of L-SNEDDS loaded with carvedilol (containing 500 mg carvedilol) was dispersed in 500 mL of distilled water. Then, MSN was added according to the target ratio in Table 1 and stirred using a magnetic stirrer at 500 rpm to obtain a stable suspension. Continuous stirring was applied to prevent MSN precipitation during spray drying. During the spray drying process, the inlet and outlet temperatures were set to 130 °C and 70 °C, respectively. The suction rate was maintained at 95%, with an airflow of 580 L/h, and the suspension feeding rate was controlled at 4.8 mL/min to ensure consistent coagulation.

### 3.4. Solubility Studies

Approximately 50 mg of carvedilol or S-SNEDDS was introduced into 5 mL of each prepared sample solution. To ensure complete equilibration, the samples were placed in a temperature-controlled shaking water bath (Daihan Scientific, Wonju, Republic of Korea) at 25 °C, and the shaking speed was set to 100 rpm and then incubated for 7 days. After incubation, the samples were centrifuged at 15,000× *g* for 10 min using an Eppendorf 5430 R centrifuge (Eppendorf, Hamburg, Germany), and the resulting supernatant was collected. The supernatant was then filtered through a 0.45 µm nylon filter and diluted with acetonitrile in preparation for carvedilol quantification.

Quantification of carvedilol was performed using high-performance liquid chromatography (HPLC) on an Agilent 1260 Infinity system (Agilent Technologies, Santa Clara, CA, USA) equipped with a 4th pump (G1311C 1260) and a variable wavelength detector (G1314B 1260). ChemStation software (version B.03.02) was used to calculate peak areas. An Inertsil ODS column (4.6 mm I.D. × 150 mm, 5 μm) was used as the stationary phase, and the oven temperature was maintained at 25 °C. The mobile phase, a mixture of 0.2% aqueous phosphoric acid and methanol (50:50, *v*/*v*), was delivered at a flow rate of 1 mL/min, and the eluate was monitored at a wavelength of 242 nm [2].

### 3.5. Dissolution Test

Release studies were conducted using the USP Dissolution Apparatus II paddle method. Distilled water (900 mL) was employed as the dissolution medium and maintained at 37 ± 0.5 °C, with the paddle rotating at 50 rpm [17]. Carvedilol alone and S-SNEDDS loaded with carvedilol (each containing the equivalent of 25 mg of carvedilol) were placed in the release apparatus using a Hanson 6TM dissolution tester (Hanson Research, Chatsworth, CA, USA). At specified time intervals, 3 mL samples were collected from the dissolution medium, filtered through a 0.45 μm nylon syringe filter, and analyzed for carvedilol content using HPLC.

### 3.6. Emulsion Droplet Size

S-SNEDDS (0.1 g equivalent to L-SNEDDS) was added to deionized water (150 mL) with gentle stirring. Then, 1 mL was taken, and the emulsion droplet size, PDI, and zeta potential were evaluated using a Zetasizer Nano ZS (Malvern Instruments, Worcestershire, UK). The measurements were taken at a wavelength of 635 nm and a scattering angle of 90°, with the temperature constant at 25 °C [40].

### 3.7. Pharmacokinetic Studies

Male Sprague Dawley rats were randomly assigned to two groups, with six rats in each group. The rats were anesthetized using ethyl ether and positioned prone on the operating table. Blood samples were obtained by inserting a heparin-coated polyethylene tube (50 IU/mL) into the right femoral artery. Carvedilol alone and S-SNEDDS (MSN 500) were administered orally in hard gelatine capsules (#9, Suheung Capsule Co., Seoul, Republic of Korea), with carvedilol dosed at 40 mg/kg and delivered alongside 1 mL of water [41].

Blood samples of 0.4 mL were collected using heparinized syringes at predetermined time points (0.25, 0.5, 0.75, 1, 1.5, 2, 3, 4, 6, 8, 12, and 24 h). The samples were centrifuged at 15,000× *g* for 15 min to obtain plasma, which was then stored at −20 °C for subsequent analysis. Each sample (120 µL) was combined with 20 µL of internal standard solution and vortexed for 2 min. Subsequently, 100 µL of 0.05 M NaHCO_3_ solution was added, followed by 2 min vertexing. Dichloromethane (1.5 mL) was added to each sample, mixed for 5 min, and centrifuged at 1500× *g* for 10 min. A 950 µL portion of the organic layer was carefully collected, transferred to a microtube, and evaporated under vacuum at 4000 g and 50 °C for 30 min. The resulting residue was reconstituted in 100 µL of mobile phase for HPLC analysis, with a 40 µL injection volume [1]. 

HPLC analysis was performed using a Hypersil ODS-2 column (4.6 mm I.D. × 250 mm, 5 µm) with a mobile phase consisting of 30 mM potassium dihydrogen phosphate buffer (pH 2.5) and acetonitrile in a 60:40 (*v*/*v*) ratio. The pH of the buffer was adjusted with phosphoric acid. The flow rate was set to 0.8 mL/min, and detection was carried out at a wavelength of 242 nm. The method demonstrated reproducibility with acceptable intraday and interday variability (R^2^ = 0.99). The WinNonlin software (version 8.1) determined pharmacokinetic parameters (Pharsight Corp., Mountain View, CA, USA) [2].

### 3.8. Physicochemical Characterization

#### 3.8.1. DSC

Each sample (3 mg) was placed in an aluminium pan for thermal characterization. The thermal behaviour of carvedilol alone and S-SNEDDS (MSN 500) was assessed using differential scanning calorimetry (DSC Q200; TA Instruments, New Castle, DE, USA). The analyses were conducted under a dry nitrogen purge, with samples heated from 60 °C to 200 °C at 10 °C/min [41].

#### 3.8.2. XRD

XRD evaluated the structural crystallinity properties of carvedilol alone and S-SNEDDS (MSN 500). Measurements were performed using an X-ray diffractometer (D/MAX-2500, Rigaku, Tokyo, Japan) with monochromatic Cu Kα radiation (λ = 1.54178 Å), 100 mA and 40 kV; the 2θ angle was from 3° to 50°; the angular increment was 0.02°/s, and at room temperature [6].

#### 3.8.3. FT-IR Spectroscopy

FT-IR spectra of carvedilol alone, S-SNEDDS (MSN 500), and MSN alone (4000–450 cm^−1^) were evaluated using Frontier (PerkinElmer, Waltham, MA, USA). The measurement range and resolution were 2000–800 cm^−1^ and 4 cm^−1^, respectively [42].

#### 3.8.4. SEM

The morphological features of carvedilol-loaded S-SNEDDS (MSN 500) and MSN alone were investigated by attaching the samples to brass stubs using double-sided adhesive carbon tape. These samples were then coated with platinum (at a rate of 6 nm/min, 15 mA) using an EmiTeck sputter coater (K575 K; Quorum Technologies, Lewes, UK) under vacuum (0.8 Pa) for 4 min. The morphological structures were analyzed using an SEM (S-4800; Hitachi, Tokyo, Japan) [7].

#### 3.8.5. Particle Size Analysis

The particle size of MSN and S-SNEDDS (MSN 500) was measured using a Mastersizer 3000 (Malvern, Worcestershire, UK). The particle size was determined based on the D50 value, which indicates that half of the particles have a diameter greater than this size and half have a diameter less than this size [43]. D10 is the particle size from which 10% of the particles are smaller. For D50, this is 50%, and for D90, it is 90%.

### 3.9. Statistical Analysis

All results are expressed as mean ± standard deviation. Statistical analysis was performed to evaluate differences between groups using Student’s *t*-test and one-way ANOVA, followed by Tukey’s post hoc test for multiple comparisons. Statistical calculations were performed using SPSS^®^ Version 26 (IBM, Armonk, NY, USA).

## 4. Conclusions

S-SNEDDS significantly enhanced solubility, dissolution rate, and oral bioavailability compared to carvedilol alone. Improved oral bioavailability was closely related to increased solubility and dissolution rate. S-SNEDDS (MSN 500) by spray dryer provided the smallest nanoemulsion particle size and showed the optimal MSN ratio to enhance solubility and dissolution rate. The physicochemical properties of S-SNEDDS were evaluated using DSC, XRD, FT-IR, and SEM. The results showed that it exhibited a smaller particle size than MSN alone, and the crystalline drug was encapsulated into MSN by changing into an amorphous substance. These results suggest that MSN can be a novel biocompatible carrier contributing to a safer and more effective delivery system.

## Figures and Tables

**Table 1:** Composition of carvedilol-loaded S-SNEDSS using MSN.

Formulation	MSN 500	MSN 750	MSN 500
Carvedilol (g)	0.10	0.10	0.10
Peceol (g)	0.25	0.25	0.25
Tween 80 (g)	0.50	0.50	0.50
Labrazol (g)	0.25	0.25	0.25
MSN (g)	0.50	0.75	1.00

**Table 2:** Pharmacokinetic parameters.

Parameters	Carvedilol Alone	S-SNEDDS
AUC (h·µg/mL)	4.17 ± 1.02	90.41 ± 5.91
C_max_ (µg/mL)	0.72 ± 0.11	11.31 ± 1.15
T_max_ (h)	2.00 ± 0.00	2.00 ± 0.00
t_1/2_ (h)	8.50 ± 2.23	11.21 ± 4.15
K_el_ (h^−1^)	0.08 ± 0.01	0.06 ± 0.01

Each value represents the mean ± S.D. (n = 6).

## Data Availability

Data are available on request due to restrictions, e.g., privacy or ethical.
